# In vitro modelling of epithelial and stromal interactions in non-malignant and malignant prostates

**DOI:** 10.1054/bjoc.1999.1029

**Published:** 2000-02

**Authors:** S H Lang, M Stower, N J Maitland

**Affiliations:** Yorkshire Cancer Research Unit, Department of Biology, University of York, Heslington, York, YO10 5YW, UK; Department of Urology, York District Hospital, Wigginton Road, York, YO31 8HE, UK

**Keywords:** prostate cancer, epithelia–stroma interactions

## Abstract

To study the effects of stromal epithelial cell interactions on prostate cancer metastasis, we have used primary human prostatic stromal cells derived from malignant and non-malignant tissues and established epithelial cell lines from normal (PNT1a and PNT2-C2) and tumour (PC-3, DU145 and LNCaP) origins. The effects of stromal cells on epithelial cell growth were studied in direct and indirect (using culture inserts) co-culture and by exposure to stromal cell-conditioned medium (assessed by MTT assay). The influence of stromal cells on epithelial cell invasion was measured using matrigel invasion chambers and on epithelial cell motility using time lapse microscopy. Results indicated that epithelial cell line growth was similarly unaffected or inhibited by stromal cells derived from malignant (*n* = 8) or non-malignant tissue (*n* = 8). In contrast, PNT2-C2 and PC-3 cells were found to be the least and the most invasive and motile epithelia respectively. Stromal cultures enhanced the invasion of both epithelial cells, but no differences were observed between the use of malignant and non-malignant tissues. All stromal cultures modestly stimulated PNT2-C2 motility but displayed a greater stimulation of PC-3 cell motility, while stromal cells derived from malignant tissue stimulated PNT2-C2 and PC-3 cell motility more than stromal cultures from non-malignant tissues. © 2000 Cancer Research Campaign


*British Journal of Cancer* (2000) **82**(4), 990–997
© 2000 Cancer Research Campaign
DOI: 10.1054/ bjoc.1999.1029, available online at http://www.idealibrary.com on IDEAL®

### In vitro modelling of epithelial and stromal interactions in non-malignant and malignant prostates

**SH Lang[1], M Stower[2] and NJ Maitland[1]**

[1]Yorkshire Cancer Research Unit, Department of Biology, University of York, Heslington, York YO10 5YW, UK; [2]Department of Urology, York District Hospital, Wigginton Road, York YO31 8HE, UK

**Summary** To study the effects of stromal epithelial cell interactions on prostate cancer metastasis, we have used primary human prostatic stromal cells derived from malignant and non-malignant tissues and established epithelial cell lines from normal (PNT1a and PNT2-C2) and tumour (PC-3, DU145 and LNCaP) origins. The effects of stromal cells on epithelial cell growth were studied in direct and indirect (using culture inserts) co-culture and by exposure to stromal cell-conditioned medium (assessed by MTT assay). The influence of stromal cells on epithelial cell invasion was measured using matrigel invasion chambers and on epithelial cell motility using time lapse microscopy. Results indicated that epithelial cell line growth was similarly unaffected or inhibited by stromal cells derived from malignant ($n = 8$) or non-malignant tissue ($n = 8$). In contrast, PNT2-C2 and PC-3 cells were found to be the least and the most invasive and motile epithelia respectively. Stromal cultures enhanced the invasion of both epithelial cells, but no differences were observed between the use of malignant and non-malignant tissues. All stromal cultures modestly stimulated PNT2-C2 motility but displayed a greater stimulation of PC-3 cell motility, while stromal cells derived from malignant tissue stimulated PNT2-C2 and PC-3 cell motility more than stromal cultures from non-malignant tissues. © 2000 Cancer Research Campaign

**Keywords**: prostate cancer; epithelia–stroma interactions

Prostate cancer is the most common form of male cancer in the Western world (Dijkman and Debruyne, 1996). It is thought that most elderly men have foci of prostate cancer but most of these tumours are latent and only a few patients will develop life-threatening disease. Individual patients can have multiple tumour foci, of which only one or a few will actually progress to a metastatic stage. At present there are no means with which to identify which tumours will progress and thus require interventive measures. Early development of prostate epithelial cells is dependent on the prostatic mesenchyme. Since tumour cells are often considered to have reverted to a more primitive developmental stage (due to their motile and invasive nature) they may also be regulated by the prostatic stroma/mesenchyme. Such a hypothesis has been discussed by Hayward et al (1997). Indeed, studies have shown that tumour-derived fibroblasts can alter the morphology of epithelial cells in vitro to a more malignant phenotype (Atula et al, 1997).

Studies investigating the interactions between prostatic stroma and epithelia have produced conflicting data. In vitro models have shown that primary cultures of prostatic stromal cells can stimulate epithelial growth derived from normal and malignant tissue (Kabalin et al, 1989) but also that prostate fibroblasts derived from normal and malignant tissue inhibit or have mixed effects on epithelial growth (Konig et al, 1987; Kooistra et al, 1995*a*; Degeorges et al, 1996). In vivo models indicate that whilst rat prostate tumour fibroblasts can stimulate LNCaP tumour cell growth, embryonic mouse fibroblasts had no effect (Camps et al, 1990; Gleave et al, 1991). Extensive studies have not been carried out with the latter model to establish whether or not tumour formation was stimulated by prostate tumour fibroblasts only. These studies did not establish whether the increased tumour formation was due to increased tumour cell proliferation or whether other factors important for metastasis were involved.

Our interest lies in looking for changes to epithelial–stromal interactions in the early stages of prostate cancer metastasis. In the light of previous work, we initially looked at the effects of stromal cell cultures on epithelial cell growth. Since prostate cancer is a very heterogeneous disease in both its presentation in the population and in its focal and cellular nature (MacIntosh et al, 1998), we decided to analyse whether or not this heterogeneity explains the conflicting results generated so far and whether any overall trends could be established. Therefore we analysed the effects of several primary stromal cultures derived from malignant and non-malignant prostate tissues on the growth of immortal prostate epithelial cell lines, also derived from malignant and non-malignant tissues. In addition we wanted to extend the study from growth assays, to investigate the effects stromal cultures had on cellular characteristics important for metastasis (invasion and motility). Such studies were devised to help understand the differences occurring between in vitro and in vivo assays.

### MATERIALS AND METHODS

### Epithelial cell line culture

PNT-1a and PNT2-C2 are normal, immortalized prostate epithelial cell lines (Berthon et al, 1995). Both are routinely cultured in RPMI-1640 (Life Technologies, Paisley, UK) culture medium supplemented with 10% fetal calf serum (FCS). Malignant prostate epithelial cell lines, PC-3, DU145, LNCaP (fast growing

*Received 15 March 1999*
*Revised 23 August 1999*
*Accepted 26 August 1999*

*Correspondence to:* SH Lang. E-mail: sh12@york.ac.uk clone) and MDA MB 321 (breast carcinoma) were obtained from the European Collection of Animal Cell Cultures (Porton Down, UK). DU145 LNCaP and MDA MB 231 cells were routinely cultured as for the normal epithelial cell lines, whilst PC-3 cells were cultured in Ham's F12 (ICN, Basingstoke, UK) culture medium supplemented with 7% FCS (Life Technologies). All cells were grown routinely in antibiotic-free media at 37°C in 5% carbon dioxide.

### Primary stromal cell culture

Stromal cultures were prepared and characterized as described before (Lang et al, 1998). Briefly, a cell pellet enriched for fibroblasts was produced from prostatic tissue (obtained by transurethral resection or prostatectomy) by collagenase digestion and differential centrifugation. Whenever possible 'tumour stroma' was derived from the excision of obvious malignant nodules from a piece of tumour tissue. The enriched stromal fraction was resuspended in stromal cell growth medium (RPMI-1640 medium supplemented with 10% FCS and 1% antibiotic/antimycotic solution) and used to produce equivalent stromal cell cultures in 75 ml tissue culture flasks and 24-well culture plates, as required. This ensured that all the stromal cell cultures were not passaged and yet could be characterized and comparative studies made.

### Stromal characterization

Cultures of stroma were prepared in 24-well plates, washed twice in phosphate-buffered saline (PBS) and then fixed in methanol–acetone (1:1) for 10 min. Cultures were blocked for 30 min with 0.3 ml Tris-buffered saline (TBS) supplemented with 1% bovine serum albumin (BSA) and 5% rabbit serum. Individual wells were incubated with either 0.3 ml TBS supplemented with 1% BSA, or 1/200 anti-vimentin (Sigma, Poole, UK), or 1/3200 anti-pan cytokeratin (Sigma), or 1/400 anti-smooth muscle actin (Sigma). After washing twice with TBS all wells were incubated with 1/300 biotin-labelled rabbit anti-mouse (Dako, High Wycombe, UK) for 30 min, followed by peroxidase streptavidin ABComplex (Dako, prepared to manufacturer's instructions) followed by 5 min with 3,3′ diaminobenzidine tetrahydrochloride (DAB; Sigma) solution.

### Collection of stromal culture conditioned medium

Confluent cultures of stromal cells were prepared in 75 ml flasks. These were washed with PBS and then incubated with 15 ml of serum-free medium Dulbecco's modified eagle's medium (DMEM)/F12 (Life Technologies) supplemented with 10 μg $ml^{-1}$ insulin, 5 μg $ml^{-1}$ transferrin and 1 ng $ml^{-1}$ selenium), for 48 h. The conditioned medium was removed, filtered (0.2 μm pore) and frozen at −20°C until required.

### Direct co-culture of epithelial and stromal cells

Confluent stromal cultures were prepared in 24-well plates, medium was aspirated and replaced with 0.5 ml of stromal cell growth media. Then 0.5 ml of an epithelial cell solution ($10^4$ cells $ml^{-1}$ prepared in stromal cell growth media) was added to each well of stromal cells and left to grow for 5 days. Control wells were established of stromal cultures alone, epithelial cells alone and tissue culture plastic alone. Each experiment was done in triplicate. Epithelial cell number was evaluated by cytokeratin enzyme-linked immunosorbent assay (ELISA) as follows. Co-cultures were washed twice in PBS and then fixed in methanol–acetone (1:1) for 10 min. Cultures were blocked for 30 min with 0.35 ml PBS supplemented with 1% BSA and 5% rabbit serum and then incubated with 0.35 ml of 1/3200 anti-pan cytokeratin (Sigma) for 1 h. Cultures were washed twice with PBS supplemented with 0.2% BSA and then incubated with 0.35 ml of 1/300 biotinylated rabbit anti-mouse (Dako) for 1 h. Cultures were washed as before, then incubated with 0.35 ml of 1/1000 streptavidin β galactosidase (Boehringer Mannheim, Lewes, UK) for 30 min, washed then incubated with 0.35 ml of 3 mM ONPG (Boehringer Mannheim) for 15 min finally 0.14 ml of 1 M $Na_2CO_3$ was added to stop the reaction. Plates were read at 405 nm. Results were blanked against control stromal cultures alone or culture plastic.

### Morphology of epithelial cells In co-culture

After 5 days growth in direct co-culture, cultures were fixed and incubated with anti-pan cytokeratin as before. They were then incubated with 1/30 (v/v) TRITC-conjugated rabbit anti-mouse (Dako) for 30 min. Cultures were then photographed using a Nikon Eclipse TE300 fluorescent microscope.

### Indirect co-culture of epithelial and stromal cells

Confluent stromal cultures were prepared in 24-well plates, medium was aspirated and replaced with 0.5 ml of stromal growth media (or RPMI-1640 supplemented with 1% FCS and 1% antibiotic/antimycotic solution). Cell culture inserts (0.4 μm Millipore, Watford, UK) were added to each well and 0.25 ml of an epithelial cell solution ($2 \times 10^4$ cells $ml^{-1}$ prepared in stromal cell media supplemented with either 10% or 1% FCS) was added to each insert and incubated for 5 days. Control wells of epithelial cells were established above tissue culture plastic alone. Each experiment was done in duplicate. Epithelial cell number was evaluated by 3-(4,5-dimethylthiazal-2-yl)-2,5-diphenyltetrazolium bromide (MTT) assay. Then 100 μl of MTT (Sigma) solution (1.5 mg $ml^{-1}$ MTT in PBS) was added to the inserts and left to incubate for 4 h, after which the inserts were removed from the stromal cell cultures and added to fresh 24-well plates. Culture media was removed from the inserts and replaced with 300 μl dimethyl sulphoxide (DMSO) (Sigma). The absorbance of the solutions was read at 570 nm.

### Effects of stromal cell conditioned media on epithelial cell growth

Cell lines were plated at 500 cells per well into 96-well plates in 0.1 ml of stromal cell conditioned medium or a control of serum-free medium (prepared as above). After plating, 0.1 ml of serum-free medium or 0.1 ml of stromal cell growth medium was added to each well as appropriate. Each experiment was done in triplicate. After 5 days growth epithelial cell numbers were evaluated by MTT assay. Then 100 μl of culture medium was removed and replaced with 50 μl MTT solution and left to incubate for 4 h, after which the culture media was removed and replaced with 150 μl DMSO. Absorbance of the solutions was read as before.

© 2000 Cancer Research Campaign 

### Invasion assay

Invasion chambers were prepared by diluting Matrigel (Becton Dickinson) 1/45 (v/v) with DMEM (Life Technologies), following the manufacturers' instructions. A total of 100 μl of diluted Matrigel was added to each cell culture insert (8 μm pore, Becton Dickinson) and left over night at 37°C. Stromal growth medium was aspirated from stromal cultures prepared in 24-well plates and replaced with 0.5 ml DMEM supplemented with 0.1% (w/v) BSA. Matrigel-coated inserts were placed over the stromal cultures. Epithelial cells were prepared in DMEM supplemented with 0.1% (w/v) BSA to a concentration of $4 \times 10^5$ cells $ml^{-1}$ and 0.25 ml of this cell suspension was added to each insert. Control wells, which contained no stromal cell cultures, were also prepared. Each experiment was carried out in duplicate. The invasion assay was left overnight, after which the inserts were removed from the wells, washed in PBS, fixed in methanol for 10 min and then stained with 0.1% (w/v) crystal violet (Sigma). Cells which had invaded to the underside of the insert were counted according to the manufacturers' instructions.

### Motility assay

Epithelial cells were plated into 25-ml flasks at a concentration of $5 \times 10^3$ cells $ml^{-1}$ in epithelial growth medium. After an overnight incubation, cells were washed in PBS and medium was replaced with 3 ml stromal conditioned medium and 3 ml epithelial growth medium. Cultures were gassed with 5% carbon dioxide and then placed onto the heated stage of a Nikon Eclipse TE300 microscope. A colony of approximately 8–16 cells was selected and images were captured with a JVC video camera, and recorded on computer using a Scion Image CG7 frame grabber (Scion Corporation, Frederick, MD, USA). Images were recorded for 8 h (120 frames were grabbed, one frame every 4 min) and then motility was scored, based on the method of Mohler et al (1988) according to Table 1.

### Statistical analysis

Results were compared using the Mann–Whitney test with a threshold for significance at $P < 0.05$.

### RESULTS

### Fibroblast characterization

Stromal cultures were characterized according to the proportion of cells in a given culture which stained for vimentin, smooth muscle α actin or cytokeratin. The results are summarized in Table 2 and an example of staining is shown in Figure 1. Stromal cells cultured from benign and malignant tissue were found to have similar characteristics, i.e. the cells were predominantly vimentin-positive and with little variation in the proportion of positive cells between different cultures. Approximately half of the cells in culture stained for smooth muscle α actin, though the ranges were much more variable, and a small percentage of cells were cytokeratin-positive. Stromal cultures, which contained a small proportion of epithelial cells were not found to significantly affect experimental results in comparison to stromal cultures without epithelia.

### Growth of prostatic epithelial cells in direct co-culture with prostatic stroma

Prostate epithelial cell lines were plated directly onto confluent stromal cultures. The cytokeratin phenotype of the epithelial cells growing on stromal cells derived from non-malignant prostate tissue is shown in Figure 2. Essentially the stromal cells form a confluent layer below (and above; see below) the epithelium. Only the cytokeratin-positive (red) epithelial cells are shown for clarity.

PNT1a cells showed a marked change in morphology from cobblestone epithelia on tissue culture plastic to very elongated, scattered fibroblast-like cells on stromal cells. PNT2-C2 cells retained colony formation on the stromal cells, but the cells in the colony were more compact and elongated. With tumour epithelial cells, PC-3 and DU145 cells showed morphology changes similar to PNT1a, although DU145 cells were the most heterogeneous in response, with > 75% of colonies adopting the 'scattered' morphology, whereas 25% of colonies had the tight morphology shown with PNT2-C2. In Figure 2, a tight colony of DU145 is shown surrounded by three scattered colonies, when plated on non-malignant stromal cells. In contrast, LNCaP cells, which produce striking multilayers on tissue culture plastic, showed changes similar to PNT2-C2, i.e. they adopted a single elongated cell layer when co-cultured with stromal cells.

These experiments were repeated on three different stromal cell cultures derived from non-malignant prostatic tissue and four derived from malignant tissue, and no observable differences were detected between the tissue types. However, 50% of the stromal cultures derived from malignant tissue resulted in co-cultures where all the epithelial cell line colonies were invasive, i.e. grew within the stroma rather than on top.

The relative ability of prostate epithelial cells to grow on different stromal cultures is shown in Figure 3. Individual cell lines showed a large range of growth responses on different stromal cells. In general, stromal cells from non-malignant and malignant tissue could be either inhibitory or stimulatory for a given epithelial cell line; however, median values indicate that on average co-cultures with stromal cells were inhibitory to cell line growth in comparison to growth on tissue culture plastic alone. No significant differences were found between the growth of any cell line on stromal cells derived from malignant tissue in comparison to that

**Table 1** Quantification of motility assays

| | | | | | | |
|---|---|---|---|---|---|---|
| Score | | 0 | 1 | 2 | 3 | 4 |
| Ruffling | | | None/little | Average | Lots | |
| Pseudopods (approx. % of cell population) | | None | 1–49% | 50% | 51–99% | 100% |
| Score | 0 | 1 | 2 | 3 | +1 | +2 |
| Translation | None | Little movement as a tight colony | Lots of movement as a tight colony | Movement as a scattered colony | < 50% of cells show individual translation | > 50% of cells show individual translation |

 *© 2000 Cancer Research Campaign*

**Table 2** Characterization of primary prostate stromal cultures

| Tissue derivation | *n* | Vimentin[a] | Smooth muscle α actin[a] | Cytokeratin[a] |
|---|---|---|---|---|
| BPH | 20 | 95% (80–100) | 50% (0–90) | 5% (0–35) |
| CaP | 14 | 95% (85–100) | 50% (0–100) | 5% (0–30) |

[a]The median value of the percentage of positively staining cells in primary cultures for a given antibody are presented (with the range of positivity for all cultures in brackets).

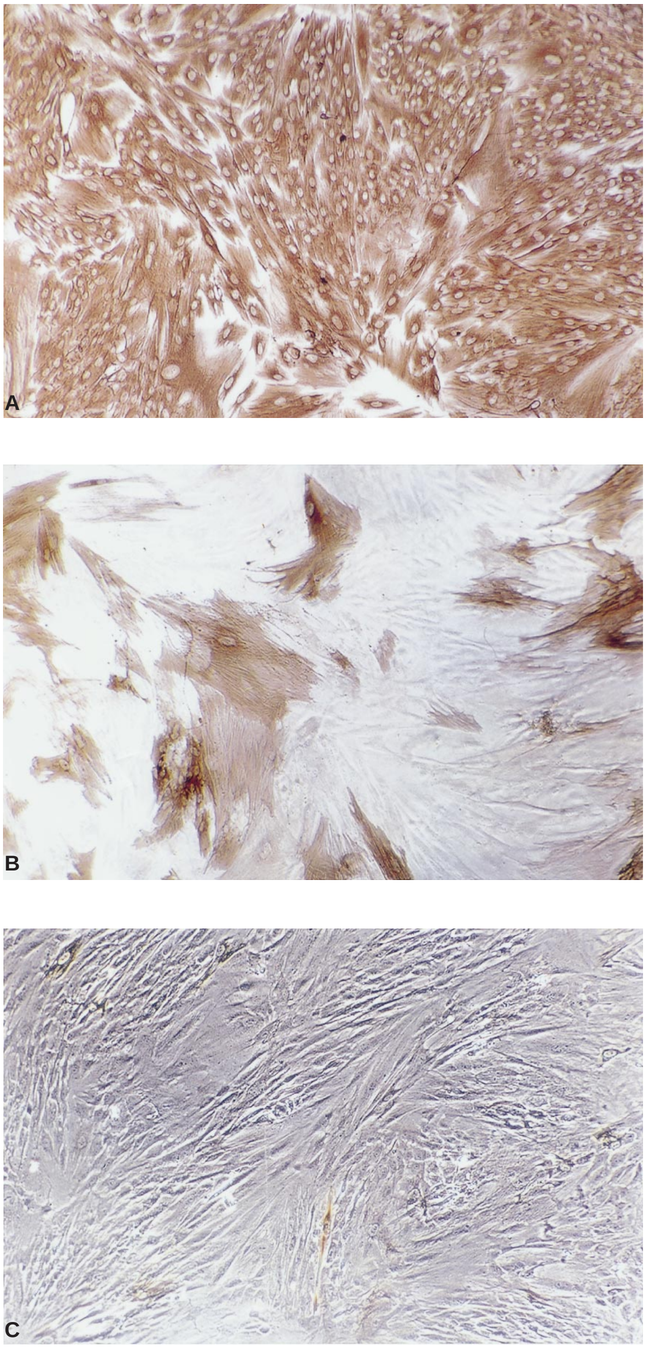

**Figure 1** Typical immunocytochemical characterization of a stromal culture derived from non-malignant tissue. The proportion of cells staining for (**A**) vimentin, (**B**) smooth muscle α actin or (**C**) cytokeratin are shown in brown derived from non-malignant tissue and no differences were found between the growth of normal epithelia and malignant epithelia on cells from either stromal type. Direct co-culture experiments of epithelia on foreskin fibroblasts showed no significant growth differences to that on prostatic stromal cells (results not shown).

### Growth of prostatic epithelial cells indirectly co-cultured with prostatic stromal cells

Using either tissue culture inserts (Figure 4) or exposure to medium conditioned by prostatic stromal cells (Figure 5), prostate epithelial cells were indirectly co-cultured with prostatic stromal cells. The results indicated that indirect stromal co-culture did not affect the growth of epithelia, and indeed predominantly caused growth inhibition. In low serum or serum-free conditions, addition of stromal factors were growth stimulatory, by up to 40% with PNT2-C2 and LNCaP cells, and up to 70% with PNT1a (median values). Again, growth responses varied considerably between different stromal cultures leading to large disparities in growth response. This was not found to correlate to the difference in cell types present in the stromal cultures. Overall we found no significant differences between the use of either normal and malignant epithelial cells or the use of stromal cells derived from malignant and non-malignant tissue sources. Exceptions to this were the growth of PC-3 cells, which was significantly more inhibited when exposed to medium conditioned by stromal cells derived from malignant tissue in comparison to that from non-malignant tissue (serum-free conditions), while PNT1a cell growth was stimulated significantly more in medium conditioned by stromal cells derived from non-malignant tissue in comparison to those derived from malignant tissue (serum-free conditions).

### Effects of stromal cultures on epithelial cell invasion

The invasion of prostate cell lines through matrigel invasion chambers was measured in the absence of stromal cultures. PC-3 and PNT2-C2 were found to be the most and the least invasive (results not shown), therefore we selected these two lines for subsequent experiments. Figure 6 shows typical results of cell line invasion in the presence of stromal cultures. In the absence of stromal cells, 0.5 PNT2-C2 cells showed invasion per average field compared to 24 PC-3 cells per average field and 118 MDA MB231 cells per average field. MDA MB 231 is an invasive breast cancer cell line and was included as a positive control. Indirect co-culture of both normal and malignant epithelial cells with stromal cultures led to an increase in their invasive ability, up to 16 and 178 cells per average field for PNT2-C2 and PC-3 cells respectively. No significant differences were measured between the effects of stromal cells derived from two malignant and two non-malignant tissues and these findings remained true when repeated on up to eight different stromal cultures of each tissue type.

*© 2000 Cancer Research Campaign* 

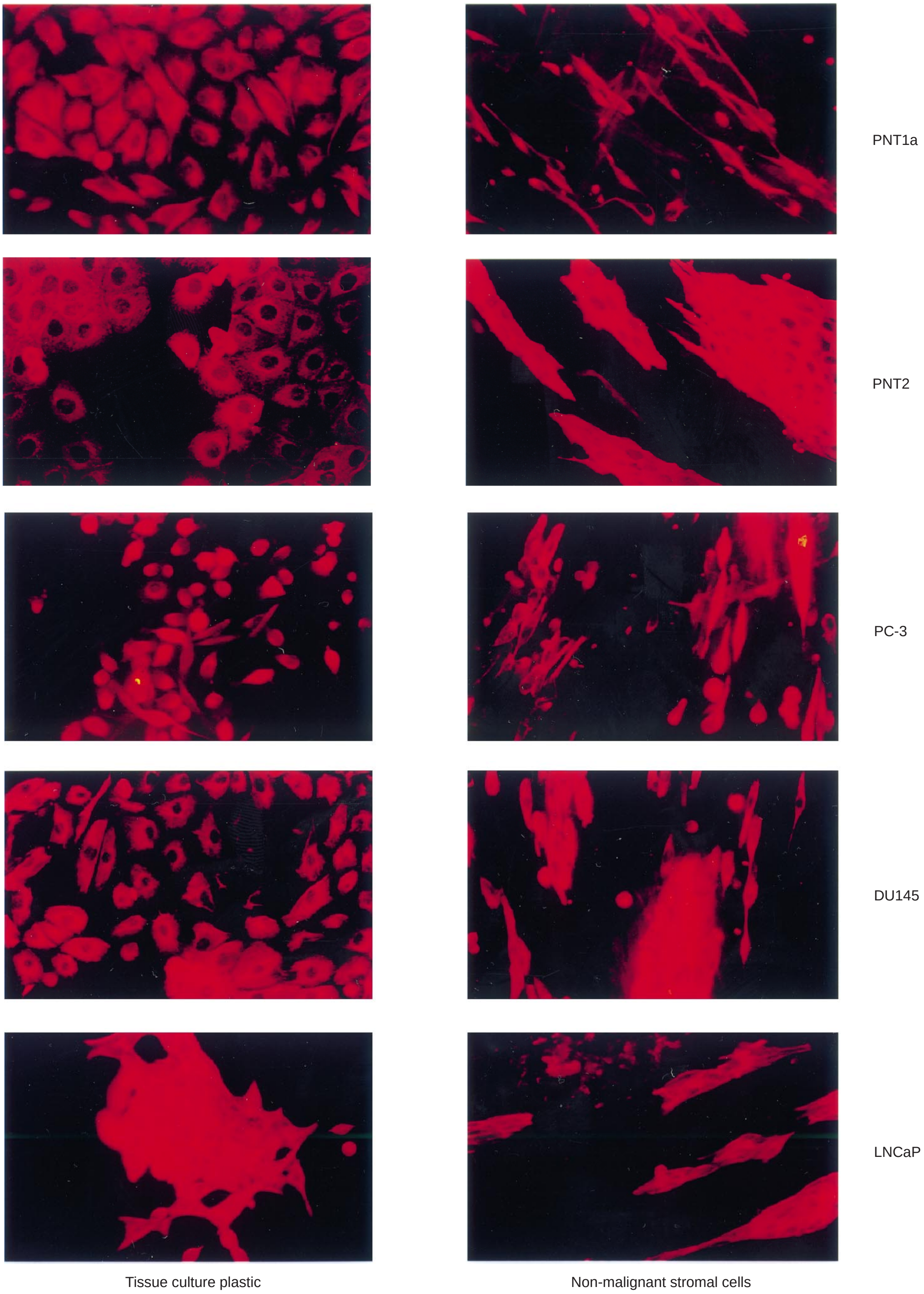

**Figure 2** Typical cytokeratin immunostaining of prostate cell lines plated onto tissue culture plastic or a stromal culture derived from non-malignant tissue. Cell lines plated on to confluent stromal cell monolayers were: PNT1a, PNT2-C2, PC-3, DU145 and LNCaP

### Effects of media conditioned by stromal cultures on epithelial cell motility

The motility of epithelial cells was graded according to the level of cell membrane ruffling, pseudopodial and translative movement. Initial experiments showed again that PNT2-C2 and PC-3 cells respectively showed the least and the most motility of the five cell lines (Table 3). Therefore these were selected for exposure to stromal cell conditioned media, and more detailed study. Exposure

*© 2000 Cancer Research Campaign*

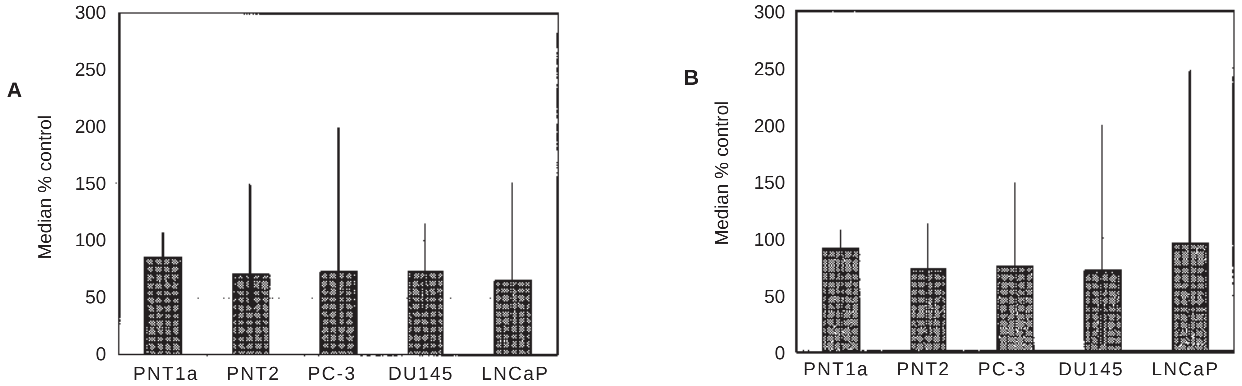

**Figure 3** Direct co-culture of prostate cell lines on stromal cultures derived from malignant (**A**) or non-malignant (**B**) prostatic tissues. After 5 days co-culture, growth was assessed by cytokeratin ELISA and compared to controls on tissue culture plastic. Each bar represents the median value of nine different stromal cultures with the range of values shown by the error bar of PNT2 C2 cells to 11/13 stromal cell conditioned media led to an increase in motility (Figure 7). Increased motility was primarily due to increased ruffling and some translative movement. The conditioned media had little effect on pseudopodial production (results not shown). Similarly 14/18 stromal cell conditioned media led to an increase in PC-3 motility (four samples caused inhibition), these increases were due primarily to pseudopodial and translative movements with ruffling being less important. Overall there was a trend for stromal cells derived from malignant tissue to stimulate motility more than that from non-malignant tissue.

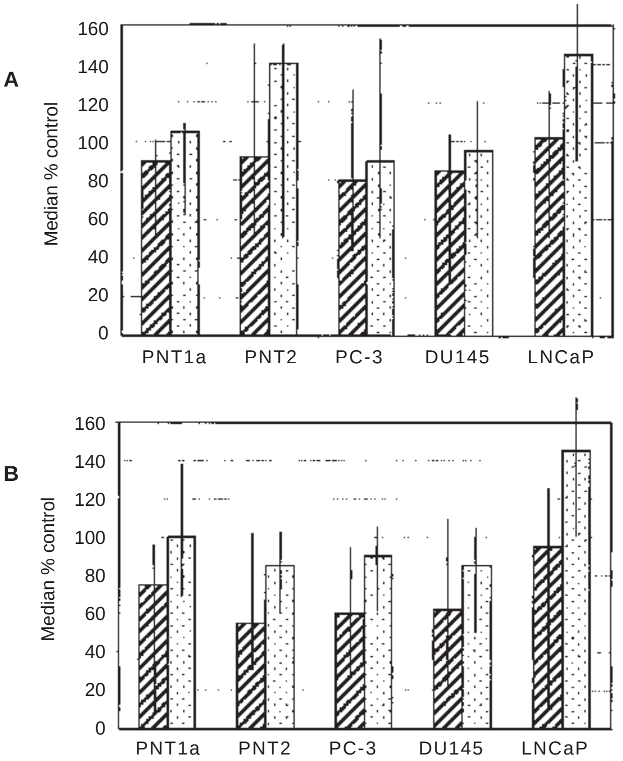

**Figure 4** Indirect co-culture of prostate cell lines with stromal cultures derived from malignant (**A**) or non-malignant (**B**) prostatic tissues. Growth was assessed after 5 days growth in either media supplemented with 10% FCS (diagonal hatched bars) or 1% FCS (stippled bars) and compared to control values. Each bar represents the median value of eight different stromal cultures with the range of values shown by the error bars

### DISCUSSION

Stromal and epithelial cells have both been implicated in the progression of prostate tumours (Atula et al, 1997; Hayward et al, 1997). To understand how stromal–epithelial cell interactions are important for normal tissue growth and how they might change in

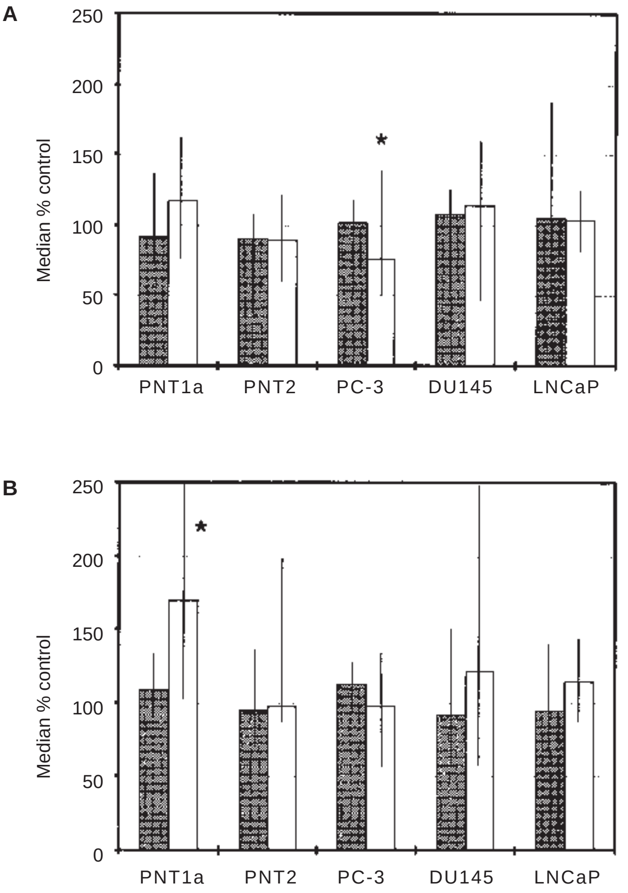

**Figure 5** Growth of prostate cell lines in the presence of media conditioned by stromal cultures derived from malignant (**A**) or non-malignant (**B**) prostatic tissues. Growth was assessed after 5 days in either media supplemented with 5% FCS (grey-filled bars) or serum free conditions (unfilled bars) and compared to control values. Each bar represents the median value of eight different stromal cultures with the range of values shown by the error bars. $^{*}P < 0.05$

*© 2000 Cancer Research Campaign*

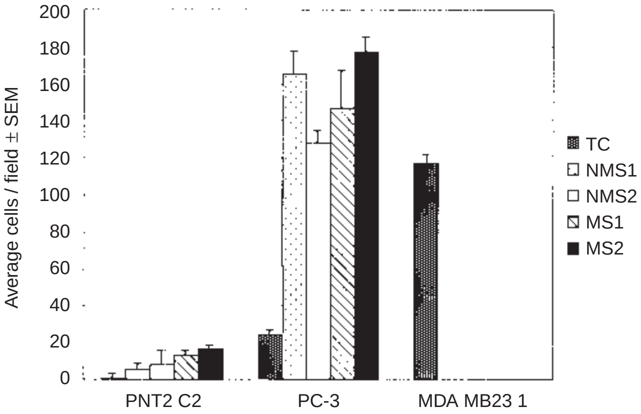

**Figure 6** Invasion of epithelial cell lines through matrigel invasion chambers in response to indirect stromal co-culture. TCP, tissue culture plastic; NMS, stroma derived from non-malignant prostate tissue; MS, stroma derived from malignant prostate tissue

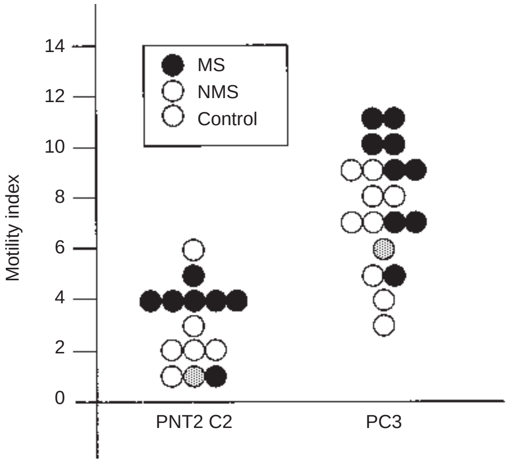

**Figure 7** The effects of stromal cell conditioned media on PNT2-C2 and PC-3 motility. Motility was graded according to the level of ruffling, translative and pseudopodial movement (Table 1) in the presence of serum free medium (control: shaded circles), media conditioned by stromal cells derived from malignant tissue (MS: filled circles) or media conditioned by stromal cells derived from non-malignant tissue (NMS: open circles)

malignant prostate tissue, we have co-cultured prostatic stromal and epithelial cells in a unique series of in vitro assays designed to investigate epithelial cell growth and metastatic ability (invasion and motility). In particular we have addressed how the source of cells (whether from malignant or non-malignant tissues) affects the results. A range of established epithelial cell lines and primary stromal tissue cultures have been used with the aim of reproducing the heterogeneity of disease presentation seen in the population. Ultimately it is hoped this approach will allow us to identify the most relevant findings and further understand the conflicting findings of other groups (Kabalin et al, 1989; Gleave et al, 1991; Kooistra et al, 1995*a*).

Overall, we found that prostate stromal cells had no significant effects on epithelial cell growth but significantly stimulated epithelial cell motility and invasion, and altered cell morphology in direct co-culture. However, the morphological changes could be reproduced with foreskin fibroblast cultures. We found no differences between the growth responses of non-malignant and malignant epithelia to stromal cultures, but non-malignant epithelial cells were less motile and invasive than malignant cells even when stimulated with stromal cultures. A heterogeneous response was only observed with the DU145 cell line. Only in the case of PNT1a cells did the conditioned medium from malignant stromal cells show a statistically significant increased growth stimulatory effect. However, this stimulation was not observed in indirect co-culture, suggesting further cross-talk between the cell types and/or the production of labile inhibitory factors which are not retained in the conditioned media.

**Table 3** Motility of prostate cell lines

| Cell line | Ruffling | Pseudopodial movement | Translative movement | Motility index |
|---|---|---|---|---|
| PNT1a | 2 | 1 | 2 | 5 |
| PNT2-C2 | 1 | 0 | 0 | 1 |
| PC-3 | 2 | 1 | 2 | 5 |
| DU145 | 2 | 1 | 1 | 4 |
| LNCaP | 2 | 1 | 1 | 4 |

Interestingly, stromal cultures derived from malignant tissue tended to stimulate the motility of epithelial cells more than stromal cells from non-malignant tissues. Similar findings have not been reported before, although research by Schor et al (1988) has shown that fibroblasts derived from cancer patients are more motile than fibroblasts derived from normal patients. This increased motility was due to the production of an autocrine motility factor. Such a factor may also stimulate epithelial motility. Increased motility was also indicated by the change in epithelial morphology when directly co-cultured with stromal cells. The epithelial cells became more fibroblastic in appearance or were more elongated, both of which are consistent with a more motile cell. Stromal cell lines have already been shown to stimulate breast cancer line motility and invasion (Heylen et al, 1998) and fibroblast-stimulated epithelial cell line invasion has been widely reported (Ito et al, 1995; Nakamura et al, 1997). Such increases in invasion were attributed to both increased expression of hepatocyte growth factor and matrix metalloproteinase production. It may be considered unusual that stromal cells can stimulate non-malignant epithelial cell invasion (though the number of actual cells invading was small), but a similar modulation of invasive capacity can be achieved with the PNT1a cells by transfection of a keratinocyte growth factor cDNA (Ropiquet et al, 1999). In this system the expression of KGF, which is expressed exclusively by fibroblasts in normal prostatic tissues, stimulated both PNT1a cell invasion through matrigel invasion chambers and the production of matrix metalloproteinase 1 and plasminogen activator, although the cells remained non-malignant in vivo. This suggests that, in vivo, other important factors are present which prevent normal epithelial cells becoming invasive, such as adhesion to the basement membrane or the type of proteolytic enzyme induced. Motility and invasion are essential characteristics for metastasis. Motility is dependent on the adhesion status of a cell (whether cell–cell or cell–matrix). The types of motility factors, adhesion molecules and proteolytic enzymes produced and induced by prostatic epithelia and stroma will be the focus of further investigations, to aid our understanding of the early events of prostate metastasis.

Initially we hypothesized that, if a prostate cancer cell were to be invasive and survive, such a cell must show some growth advantage

 *© 2000 Cancer Research Campaign*

over normal cells when in direct contact (or co-culture) with stromal cells. However, the results presented here and those found previously (Lang et al, 1998) do not support this hypothesis. Our results, using a mixed population of stromal cells, would agree with both the theories that fibroblasts can stimulate (Kabalin et al, 1989; Gleave et al, 1991) or inhibit (Kooistra et al, 1995*a*; Degeorges et al, 1996) epithelial cell growth when compared to growth on tissue culture plastic. However, the majority of stromal cultures had no effect on, or inhibited epithelial cell growth. Characterization of 34 stromal cultures indicated that they were primarily composed of fibroblasts (95%), based on an unequivocal immunostain for vimentin. Many of the vimentin-positive cells also expressed smooth muscle α actin (50%). While epithelial cells in primary cultures from human prostate can express low levels of vimentin (Berthon et al, 1995), the epithelial content of the stromal cultures was assessed both on morphology and on the strong staining with cytokeratin. No significant changes in the composition were noted between stromal cells derived from malignant and non-malignant tissues. Earlier work has indicated that the proportion of smooth muscle decreases in the stroma of tumour samples (Hayward et al, 1997) but we did not find significant differences in the cell composition of primary stromal cultures, even of stromal cultures from poorly differentiated tissue. Stromal cultures were used which were unpassaged, therefore giving rise to a mixed population of cells, and while this may be considered ill-defined, we wished to use this as a model to mimic the cell populations present in the patient and also to avoid using later passaged cultures whose characteristics and growth stimulating effects become altered (Kooistra et al, 1995*a*, 1995*b*; Pasternack et al, 1997). Further investigation is required to isolate primary cultures of smooth muscle cells or fibroblasts alone and analyse their effects on epithelial cell growth. Our results also demonstrated a wide range of epithelial cell growth responses to stromal cells (stimulatory and inhibitory). This is not unusual, and has been reported for other tissue models (van Roozendal et al, 1996). Our results represent cells from many patients rather than one or two cell lines and thus provide a useful comparison, despite the obvious heterogeneity.

However all in vitro experiments have to be critically analysed, since many parameters will affect the results. These include the presence of serum, the cell density, derivation and age of cells. Further experiments aim to extend the growth studies, to take into account the potential importance of patient age, androgen dependency and tumour stage. Despite the expected and observed variability, the results presented suggest that the fibroblast stimulation of tumour formation in vivo may not be due to growth proliferation but to the stimulation of motility and invasion of epithelial cell lines. Such characteristics were not considered by the earlier experiments of Gleave et al (1991) and Camps et al (1990), but should now be applied to such models. Such ideas were addressed by Noel et al (1998) who demonstrated that the fibroblast-stimulated growth of breast tumours required metallo-proteinases.

In summary, epithelial cell motility and invasion showed the greatest disparity between normal and tumour stromal cell cultures. Primary prostatic stromal cultures derived from malignant tissue increased epithelial cell motility more than stroma derived from non-malignant tissue. Stromal cultures had no effect or inhibited epithelial cell line growth and no differences were observed between the use of normal or malignant cells. These findings will be further explored with the ultimate aim of trying to produce markers which will identify progressive prostate tumours.

### ACKNOWLEDGEMENTS

We are grateful to the Yorkshire Cancer Research for funding this project and many thanks to C Hyde and K Ramsay for their technical assistance.

### REFERENCES

Atula S, Grenman R and Syrjänen S (1997) Fibroblasts can modulate the phenotype of malignant epithelial cells in vitro. *Exp Cell Res* **235:** 180–187

Berthon P, Cussenot O, Hopwood L, Le Duc A and Maitland NJ (1995) Functional expression of SV40 in normal human prostatic epithelial and fibroblastic cells: differentiation pattern of non-tumorigenic cell lines. *Int J Oncol* **6**: 333–343

Camps JL, Chang SM, Hsu TC, MR, Hong SK, HE, Von Eschenbach AC and Chung LWK (1990) Fibroblast-mediated acceleration of human epithelial tumor growth in vivo. *Proc Natl Acad Sci USA* **87**: 75–79

Degeorges A, Tatoud R, Fauvel-Lafeve F, Podgorniak MP, Millot G, De Cremoux P and Calvo F (1996) Stromal cells from human benign prostate hyperplasia produce a growth-inhibitory factor for LNCaP prostate cancer cells, identified as interleukin-6. *Int J Cancer* **68**: 207–214

Dijkman GA and Debruyne FMJ (1996) Epidemiology of prostate cancer. *Eur Urol* **30**: 281–295

Gleave M, Hsieh J-T, Gao C, Von Eschenbach AC and Chung LWK (1991) Acceleration of human prostate cancer growth in vivo by factors produced by prostate and bone fibroblasts. *Cancer Res* **51**: 3753–3761

Hayward SW, Rosen MA and Cunha GR (1997) Stromal–epithelial interactions in the normal and neoplastic prostate. *Br J Urol* **79**: 18–26

Heylen N, Baurain R, Remacle C and Trouet A (1998) Effect of MRC-5 fibroblast conditioned medium on breast cancer cell motility and invasion in vitro. *Clin Exp Metastasis* **16**: 193–203

Ito A, Nakajima S, Sasaguri Y, Nagase H and Mori Y (1995) Co-culture of human breast adenocarcinoma MCF-7 cells and human dermal fibroblasts enhances the production of matrix metalloproteinases 1, 2 and 3 in fibroblasts. *Br J Cancer* **71**: 1039–1045

Kabalin JN, Peehl DM and Stamey TA (1989) Clonal growth of human prostatic epithelial cells is stimulated by fibroblasts. *Prostate* **14**: 251–263

König JJ, Romijn JC and Schröder FH (1987) Prostatic epithelium inhibiting factor (PEIF): organ specificity and production by prostatic fibroblasts. *Urol Res* **15**: 145–149

Kooistra A, Van Den Eijnden-Van Raaij AJM, Klaij IA, Romijn JC and Schröder FH (1995*a*) Stromal inhibition of prostatic epithelial cell proliferation not mediated by transforming growth factor beta. *Br J Cancer* **72**: 427–434

Kooistra A, Elissen NMJ, Konig JJ, Vermey M, van Der Kast ThH, Romijn JC and Schroder FH (1995*b*) Immunocytochemical characterisation of explant cultures of human prostatic stromal cells. *Prostate* **27**: 42–49

Lang SH, Clarke NW, George NJR, Allen TD and Testa NG (1998) Interaction of prostate epithelial cells from benign and malignant tumour tissue with bone-marrow stroma. *Prostate* **34**: 203–213

Macintosh CA, Stower M, Reid N and Maitland NJ (1998) Precise microdissection of human prostate cancers reveals genotypic heterogeneity. *Cancer Res* **58**: 23–28

Mohler JL, Partin AW, Isaacs JT and Coffey DS (1988) Metastatic potential prediction by a visual grading system of cell motility: prospective validation in the Dunning R-3327 prostatic adenocarcinoma model. *Cancer Res* **48**: 4312–4317

Nakamura T, Matsumoto K, Kiritoshi A, Tano Y and Nakamura T (1997) Induction of hepatocyte growth factor in fibroblasts by tumour-derived factors affects invasive growth of tumour cells: in vitro analysis of tumour–stromal interactions. *Cancer Res* **57**: 3305–3313

Noel A, Hajitou A, L'Hoir C, Maquoi E, Baramova E, Lewalle JM, Remacle A, Kebers F, Brown P, Calberg-Bacq CM and Foidart JM (1998) Inhibition of stromal matrix metalloproteases: effects on breast-tumor promotion by fibroblasts. *Int J Cancer* **76**: 267–273

Pasternack M, Jr, Liu XL, Goodman RA and Rannels DE (1997) Regulated stimulation of epithelial cell DNA synthesis by fibroblast-derived mediators. *Am J Physiol Lung Cell Mol Physiol* **272**: L619–630

Ropiquet F, Hugenin S, Villette J-M, Ronfle V, Le Brun G, Maitland NJ, Cussenot O, Fiet J and Berthon P (1999) FGF7/KGF triggers cell transformation and invasion on immortalised human prostatic epithelial PNT1A cells. *Int J Cancer* **82**: 237–243

Schor SL, Schor AM, Grey AM and Rushton G (1988) Foetal and cancer patient fibroblasts produce an autocrine migration-stimulating factor not made by normal adult cells. *J Cell Sci* **90**: 391–399

Van Roozendaal KEP, Klijn JGM, Van Ooijen B, Claassen C, Eggermont AMM, Henzen-Logmans SC and Foekens JA (1996) Differential regulation of breast tumor cell proliferation by stromal fibroblasts of various breast tissue sources. *Int J Cancer* **65**: 120–125

© 2000 Cancer Research Campaign 
